# Diet-Derived Fatty Acids, Brain Inflammation, and Mental Health

**DOI:** 10.3389/fnins.2019.00265

**Published:** 2019-03-26

**Authors:** Helen M. Melo, Luís Eduardo Santos, Sergio T. Ferreira

**Affiliations:** ^1^Institute of Medical Biochemistry Leopoldo de Meis, Federal University of Rio de Janeiro, Rio de Janeiro, Brazil; ^2^Institute of Biophysics Carlos Chagas Filho, Federal University of Rio de Janeiro, Rio de Janeiro, Brazil

**Keywords:** fatty acids, high-fat diet, Western diet, mood disorders, neuroinflammation

## Abstract

Western societies experienced drastic changes in eating habits during the past century. The modern nutritional profile, typically rich in saturated fats and refined sugars, is recognized as a major contributing factor, along with reduced physical activity, to the current epidemics of metabolic disorders, notably obesity and diabetes. Alongside these conditions, recent years have witnessed a gradual and significant increase in prevalence of brain diseases, particularly mood disorders. While substantial clinical/epidemiological evidence supports a correlation between metabolic and neuropsychiatric disorders, the mechanisms of pathogenesis in the latter are often multifactorial and causal links have been hard to establish. Neuroinflammation stands out as a hallmark feature of brain disorders that may be linked to peripheral metabolic dyshomeostasis caused by an unhealthy diet. Dietary fatty acids are of particular interest, as they may play a dual role, both as a component of high-calorie obesogenic diets and as signaling molecules involved in inflammatory responses. Here, we review current literature connecting diet-related nutritional imbalance and neuropsychiatric disorders, focusing on the role of dietary fatty acids as signaling molecules directly relevant to inflammatory processes and to neuronal function.

## Introduction

Western society experienced a marked nutritional transition during the past century. Multiple factors, stemming primarily from the industrial revolution and mass urbanization, have driven the nutritional profile of the population toward increased consumption of processed and animal-derived foods, saturated fats and refined sugars, while reducing the intake of vegetables, fruits, fibers, and fish (Popkin and Gordon-Larsen, 2004; Popkin et al., 2012). Moreover, these altered eating habits have been associated with larger portion sizes and reduced energy expenditure, making modern lifestyle highly obesogenic (Bray and Popkin, 1998; Hill and Peters, 1998; Kopelman, 2000; Berthoud, 2012). During the same time span, the prevalence of mood disorders has increased significantly in Western countries, even when accounting for evolving diagnostic criteria and other confounding factors (Hagnell, 1989; Hidaka, 2012). As mechanisms linking diet to mental health become better understood, accumulating evidence suggests that the modern/Western diet may be one of the drivers of this increase (Jacka et al., 2010; O’Neil et al., 2014).

To address this link, the nascent field of “nutritional psychiatry” (Logan and Jacka, 2014) focuses on clinical studies examining the impact of both isolated nutrients and overall quality of diets on the incidence and progression of prevalent psychiatric conditions, most often anxiety and mood disorders. In parallel, the field strives to overcome challenges associated with the combined complexities of human diet and behavior, both of which are difficult to control and evaluate consistently (Jacka, 2017).

Although it is not disputed that the modern lifestyle and nutritional behavior promote a surplus of energy and its storage in the form of expanding adipose tissue (Spiegelman and Flier, 2001), the exact relevance of macronutrient composition–the balance of protein, carbohydrate and fat—to body weight regulation remains under discussion. While some authors argue for a disproportionate contribution of a single type of nutrient to the development of obesity and metabolic disorders, others sustain that such pathologies are not significantly dependent on diet composition, resting instead squarely on a positive energy balance.

Bray and Popkin (2014), for instance, have suggested that increased intake of carbohydrates—mainly in the form of glucose/fructose-sweetened beverages, a primary source of added sugars in modern society—is a key driver of the modern pandemic of obesity and metabolic conditions (Bray et al., 2004; Bray and Popkin, 2014). Indeed, numerous meta-analyses show a positive correlation between sugar consumption and increased risk of insulin resistance, non-alcoholic fatty liver disease (NAFLD), obesity and type 2 diabetes (Ludwig et al., 2001; Bray et al., 2004; Malik et al., 2006, 2010; Montonen et al., 2007; Ouyang et al., 2008; Bray and Popkin, 2014; Mosca et al., 2017). Although such correlations have not been consistently demonstrated when total energy intake is controlled, authors argue that added calories obtained from sugar-sweetened beverages tend to not be compensated elsewhere, as they do not effectively suppress intake of other calories. Mechanistically, increased fructose uptake and metabolism in the liver stimulates *de novo* lipogenesis (DNL), which results in increased intra-hepatic lipid content, leading to increased production and secretion of very low-density lipoprotein and triglycerides. In the long term, these alterations may result in increased fat storage in visceral adipose tissue and ectopic lipid deposition in tissues such as muscle, further contributing to insulin resistance (Lê et al., 2009; Stanhope et al., 2009; Stanhope, 2016; Mock et al., 2017).

On the other hand, as highlighted by Khan and Sievenpiper (2016), more recent trends of reduced sugar intake by United States adults have not been accompanied by a reduction in obesity and metabolic disorders. In addition, as mentioned above, most controlled trials using isocaloric diets have not shown a specific contribution of any type of nutrient to obesity, suggesting total energy content is the most relevant variable (Kahn and Sievenpiper, 2014; Khan and Sievenpiper, 2016).

Regarding the role of fat intake, Hu et al. (2018) recently published a compelling report comparing the long-term effects of 29 types of diet, with varying proportions of fat, carbohydrates and protein, on five different mouse strains. Surprisingly, they found fat content in a diet to be the only factor involved in increased energy intake and adiposity. This observation was explained by a hedonic drive linked to fat, but they did not observe a similar effect with sugar (Hu et al., 2018). The fact that a certain diet composition may drive higher energy intake independent of the diet’s own energy density adds an additional layer of complexity to the field, particularly in the design and execution of clinical trials.

Hu et al. (2018) present an interesting discussion on the translatability of this finding, and the feasibility of performing an equivalent study in humans. However, be it due to its higher energy content compared to other nutrients, to its reward value that drives increased caloric intake, or to specific but not yet fully understood signaling and metabolic dysregulation, the impact of increased fat consumption on the development of diet-related diseases has been well documented over the years (for an excellent recent review, see Ludwig et al., 2018). Although controversy remains on the relevance of total fat intake toward body fat accumulation, with several studies indicating no causal relationship (Curb and Marcus, 1991; Willett, 1998, 2002; Vergnaud et al., 2013), excessive energy intake from dietary fat is established as an important factor to increased adiposity (Horton et al., 1995; Bray and Popkin, 1998).

The fantastic remodeling capacity of adipose tissue allows for adipocyte hypertrophy and hyperplasia in response to nutrient availability and energy surplus. However, under pathological conditions, the need for adaptation exceeds the capacity of the tissue. Hypoxia and adipocyte cell death result in macrophage recruitment and polarization, increasing inflammatory markers, cytokine and chemokine secretion, and dysregulation in free fatty acid (FFA) fluxes (Sun et al., 2011).

Increased circulating FFAs and proinflammatory factors are also central to insulin resistance and deregulation of glucose homeostasis, the core aspects of type 2 diabetes mellitus (T2DM) (Greenberg and Obin, 2006). Obesity and T2DM currently affect a large portion of the world population and are considered a global epidemics, with obesity as the leading risk factor for T2DM (Barnes, 2011).

Notably, the fatty acid composition of diets has been shown to impact their obesogenic profile and overall toxicity. Particularly, enrichment in saturated fatty acids (SFAs) results in a diet that induces greater accumulation of body fat and lower satiety than diets enriched in polyunsaturated fatty acids (PUFAs) (Lawton et al., 2000; Piers et al., 2003; Moussavi et al., 2008; Phillips et al., 2012). Moreover, excessive SFA consumption was shown to increase SFAs in the circulation, increase expression of genes involved in inflammatory processes in adipose tissue, reduce insulin sensitivity and increase intrahepatic triglyceride content in humans (Vessby et al., 2001; van Dijk et al., 2009; Rosqvist et al., 2014).

Long-term longitudinal studies have linked a low intake of PUFAs and a high intake of cholesterol and SFAs to increased risk of impaired cognitive function and development of dementia, including Alzheimer’s disease (Kalmijn et al., 1997; Morris et al., 2003a,b, 2004; Barnard et al., 2014; Reichelt et al., 2017). In this context, it has been suggested that metabolic imbalance caused by high-fat diets and a sedentary lifestyle constitutes an important AD risk factor, particularly due to its association with higher levels of plasma FFAs, chronic low grade inflammation, insulin resistance and T2DM (De Felice, 2013). Further, whereas moderate intake of PUFAs at midlife appears to decrease the risk of dementia in aging (Laitinen et al., 2006), saturated and trans-unsaturated fat consumption have been found to be positively associated with increased risk of AD (Morris et al., 2003b). Adherence to a Mediterranean diet and frequent consumption of fruits and vegetables, fish, and ω-3 (n-3 PUFA) rich oils has been proposed as a factor capable of preventing AD and dementia (Scarmeas et al., 2006; Barberger-Gateau et al., 2007, 2011). Moreover, cognitive performance in elderly people (65–90 years old), free from significant cognitive impairment, was better in subjects having high intakes of vegetables, fruits, and vitamins and lower intakes of monounsaturated fatty acids, SFAs, and cholesterol (Ortega et al., 1997).

A close relationship exists between metabolic syndrome, T2DM and brain dysfunction, encompassing both mood and cognitive disorders (Ott et al., 1996; De Felice, 2013; Santos et al., 2016; Rebolledo-Solleiro et al., 2017). The mechanisms underlying this connection appear largely based on neuroinflammation and dysregulated brain insulin signaling, both of which can result from nutritional imbalance (reviewed in Luchsinger, 2012; De Felice and Ferreira, 2014; Holt et al., 2014; Sevilla-González et al., 2017).

In the following sections, we focus on data available on the connection between dietary fatty acids and their potential role in mental health, particularly in depressive disorders. We explore how, due to their potential as modulators of neuroinflammation and insulin signaling, fatty acids may be key to the interplay between diet and mental health. We also discuss some of the more recent work exploring how the dopaminergic system, increasingly implicated in the pathophysiology of depression, may be affected by dietary choices.

## Human Populational Studies

While numerous observational studies have been carried out, randomized controlled trials (RCTs) on the relationship between diet and mood disorders are comparatively rare. A meta-analysis conducted in 2014 (Lai et al., 2014) examined links between dietary patterns and depression, and found 20 observational studies meeting inclusion criteria, but only one RCT. These authors concluded that ‘healthy’ diets, including a high intake of fruit, vegetables, fish and whole grains, were inversely correlated with depression. Around the same time, another meta-analysis carried out by Psaltopoulou et al. (2013) reached similar conclusions while examining nine observational studies that had depression as the main outcome and eight studies evaluating cognitive function. They found a significant association between adherence to the Mediterranean diet – a diet pattern similar to what Lai et al. (2014) classified as healthy – and lower rates of both depression and cognitive impairment.

Since humans do not typically consume any single type of food in isolation, studies with isolated nutrients, such as fatty acids, are not only difficult to perform, but also trade potential relevance for increased power. Thus, to approach questions dealing with the roles of specific nutrients, authors often rely on supplementation or on observational studies to seek correlations between study outcomes and specific biomarkers reflecting a nutrient’s level of intake or metabolism. Interestingly, a meta-analysis of 13 randomized placebo-controlled trials enrolling a total of 1,233 patients with major depressive disorder (MDD) demonstrated a beneficial effect of omega-3 PUFA supplementation on depressive symptoms (Mocking et al., 2016), with a larger effect at higher doses and in patients being simultaneously treated with antidepressants, suggesting a potential adjuvant role of omega-3 fatty acids in MDD treatment.

Results from several other meta-analyses and epidemiological studies suggest that reduced levels of PUFAs could be involved in the pathogenesis of cognitive and mood disorders, and may be therapeutic targets in those diseases. A meta-analysis of 14 studies found that subjects with depressive symptoms or social anxiety disorders had lower circulating levels of the n-3 PUFAs, eicosapentaenoic acid, 20:5n-3 (EPA) and docosahexaenoic acid, 22:6n-3 (DHA), and/or higher levels of the n-6 PUFA, arachidonic acid, 20:4n-6 (ARA), than control subjects (Lin et al., 2010). In addition, post-mortem analysis of orbitofrontal and prefrontal cortex of patients with major depression showed lower DHA levels compared to controls (McNamara et al., 2007, 2013). Moreover, a recent 7-year follow-up study of 69 young individuals with an ultra-high risk phenotype for psychosis demonstrated that lower levels of EPA and/or DHA, and higher n-6/n-3 PUFA ratio in the phosphatidylethanolamine fraction of erythrocyte membranes, specifically predicted mood disorders (in this cohort, 24 patients received a diagnosis of MDD and 2 of bipolar disorder during the follow-up period; Berger et al., 2017). Altogether, these and other studies implicate PUFAs in the pathogenesis of mood and cognitive disorders, providing a basis for nutritional psychiatry approaches in these highly prevalent and incapacitating diseases.

## High-Fat Diet in Rodent Models

Many of the current inferences on the impact of HFD on human health have been based on or influenced by studies in animal models, mostly rodents. As in humans, fat-enriched diets induce rapid weight gain and metabolic alterations in animal models. Although the term ‘high-fat diet’ is widely used to describe studies in which fat corresponds to the highest proportion of energy intake, that percentage may range from 20 to 60% of total energy intake and diet composition may include animal-derived fats or plant oils. Moreover, the composition of the control diet is often not standardized, with non-purified chow being used as a control and some studies omitting to mention the composition of the control diet altogether (Buettner et al., 2007; Hariri and Thibault, 2010). Further, the age at which exposure to HFD is initiated is also variable among studies. Lack of standardization in studies under the HFD umbrella leads to great variability in observed outcomes and difficulty in establishing comparisons between studies.

Despite the different protocols, some of the effects of excess fat in the diet appear to be central and robust in mice. In a landmark report by Xu et al. (2003), for instance, obesity induced in C57BL/6J mice by long-term exposure to a HFD (containing up to 60% calories from fat) produced increases in adipocyte number and size, body weight, fasting blood glucose levels, and induced hyperinsulinemia (Xu et al., 2003). Those metabolic changes were later shown to occur even after a short period of fat-enriched diet consumption (Lee et al., 2011).

Hotamisligil et al. (1993) demonstrated a central role of TNF-α in diabetes and obesity-induced insulin resistance, using db/db, ob/ob, tub/tub, and fa/fa mice, genetic models of metabolic disorders (recently reviewed in Kleinert et al., 2018). In those mice, Hotamisligil et al. (1993) showed that increased expression of TNF-α in adipocytes as well as high levels of this cytokine in the circulation resulted in insulin resistance. In line with those findings, Xu et al. (2003) went on to show that excessive macrophage recruitment and upregulated expression of ADAM8, MIP-1α, MCP-1, MAC-1, F4/80, and CD68 in white adipose tissue contribute to the establishment of chronic inflammation and increased production and release of pro-inflammatory cytokines, notably TNF-α and IL-6, into the circulation. These results were later corroborated by others, and positioned obesity-induced inflammation into a broader picture (Hotamisligil et al., 1993; Wellen and Hotamisligil, 2003; Xu et al., 2003; Lumeng et al., 2007; Eder et al., 2009).

The HFD mouse model has been instrumental in dissecting the molecular mechanisms involved in FFA-induced T2DM. It was first shown in humans that excessive FFAs in the circulation inhibit insulin signaling and glucose metabolism in several tissues, such as adipocytes, liver, and muscle. Excessive FFAs were shown to reduce muscle glucose transport and metabolism via decreased GLUT4 translocation to the plasma membrane (Roden et al., 1996) and to inhibit insulin signaling by increasing IRS-1 serine phosphorylation and reducing insulin-stimulated PI3-kinase activity (Goodyear et al., 1995; Dresner et al., 1999). The important role of inflammatory responses in this process, which culminate in the activation of stress kinases such as JNK and IKKβ, which in turn target IRS, was described in HFD mouse models (Yuan et al., 2001; Hirosumi et al., 2002; Arkan et al., 2005).

Increased saturated FFAs, observed in obesity and high fat intake models, have an intrinsic pro-inflammatory potential that impacts important cell functions. Fatty acids may activate Toll-like receptor 4 (TLR4) signaling in adipocytes and macrophages and induce inflammatory signaling (Shi et al., 2006), and mice lacking TLR4 were shown to be protected against high-fat diet-induced obesity and insulin resistance (Poggi et al., 2007; Davis et al., 2008). TLRs are a family of type I transmembrane receptors that recognize a variety of microbial danger-associated molecular patterns (DAMPs) and pathogen-associated molecular patterns (PAMPs) and orchestrate an intracellular signaling response, playing an important role in infectious and inflammatory disorders. Amongst at least 13 members of TLRs described in mammals, TLR2 and TLR4 are best characterized in terms of their involvement in the immune response (Fessler et al., 2009). TLR4 plays a critical role in the innate immune system by activating MyD88-dependent and MyD88-independent proinflammatory signaling pathways, as well as the NFκB response (Lu et al., 2008).

Mood disorders cannot be fully reproduced in rodent models. In addition to their incompletely understood etiology, they involve symptoms that may not exist outside of the human experience, such as inappropriate guilt and suicidality (Krishnan and Nestler, 2011). However, rodent models may exhibit depressive-like symptoms, such as behavioral correlates of hopelessness or anhedonia. Recent data on rats and mice fed HFD suggest a positive association between HFD and such depressed phenotypes (Yang et al., 2016; Arcego et al., 2018; Hassan et al., 2018), which may be causally linked to diet-induced inflammatory processes, as discussed below.

## Saturated Fatty Acids and Neuroinflammation: Possible Links to Mood Disorders

Microglial cells respond rapidly to pathological changes in the brain, altering their morphology and phagocytic behavior, and increasing cytotoxic responses by secreting NO, proteases and cytokines, such as TNF-α and IL-1β (Kreutzberg, 1996). SFAs, such as palmitic acid, have been shown to induce activation of TLR4 receptors in hypothalamic microglia and to stimulate cytokine release (Valdearcos et al., 2014), indicating a potential mechanism by which HFD leads to brain inflammation. Notably, the hippocampus—a key brain region involved not only in learning and memory but also in depression and the effect of antidepressants—is vulnerable to altered levels of IL-1β, IL-6, and TNF-α, as these cytokines have important roles in synaptic plasticity and may inhibit neurogenesis (Sheline, 2011; Calabrese et al., 2014).

Microglia and astrocytes are essential to normal synaptic function. Synaptic pruning by microglia is essential to synaptic maturation and neurotransmission (Paolicelli et al., 2011), while astrocytes hold important metabolic and plasticity functions (Beattie et al., 2002; Singh and Abraham, 2017). Importantly, HFD-induced depressive-like behavior in rodents, as well as cognitive impairment, has been associated with brain inflammation. For instance, Dutheil et al. (2016) showed that, in addition to the classical metabolic alterations, rats fed an HFD (60% of calories as fat) for 16 weeks show anhedonic behavior, which presents alongside insulin signaling impairment and increased levels of cytokines such as IL-6, IL-1β, and TNF-α in the hippocampus. In turn, mice exposed to long term HFD were shown to have spatial memory deficits in the Morris water maze, with increased serum and hippocampal levels of TNF-α and presence of activated microglia in the hippocampus, as well as reduced dendritic branching and complexity (Heyward et al., 2012; Jeon et al., 2012).

In line with the structural similarity between SFAs and the lipid portion of bacterial lipopolysaccharide (LPS), several lines of evidence suggest that SFAs act as ligands of TLRs. *In vitro* experiments have shown that SFAs activate TLR2 to induce an inflammatory response (Erridge and Samani, 2009; Huang et al., 2012), and numerous reports have linked SFAs to TLR4-mediated signaling pathways in immune cells (Park et al., 2009; Rogero and Calder, 2018). Using both the BV-2 microglial cell line and primary microglial cultures, Wang et al. (2012) demonstrated that palmitic acid and stearic acid induce a reactive microglial phenotype and increase levels of inflammatory markers in a TLR4-dependent manner. The SFAs, lauric, palmitic, and stearic acids, but not unsaturated fatty acids or PUFAs, were shown to induce NF-κB activation and expression of COX-2 and other inflammatory markers in macrophages, effects inhibited in dominant-negative TLR4 cells (Lee et al., 2001). Further, the liver secretory protein fetuin-A (FetA) has been suggested as an adaptor protein between FFAs and TLR4 activation, connecting FFAs to TLR-mediated inflammation (Pal et al., 2012). Importantly, however, the role of TLRs as SFA receptors is still a matter of debate. The most recent challenge to this notion was a compelling report by Lancaster et al. (2018) suggesting that SFAs are not direct ligands of TLR4 in macrophages, but instead contribute to pro-inflammatory signaling by altering lipid metabolism in these cells. They reconcile these results with past literature findings by showing that, despite not being a direct target, TLR4-dependent priming is a requirement for SFA-induced inflammatory signaling.

Inflammation has emerged as an important factor in mood disorders. Patients presenting mood disorders show elevated plasma levels of cytokines such as TNF-α, IL-6, and IL-1β, as well as increased expression of inflammatory markers in blood cells (reviewed by Mechawar and Savitz, 2016). Increased consumption of high fat diet is related to depressive-like behavior and emotional disorders in mice (Wang et al., 2017; Arcego et al., 2018; Vagena et al., 2018; Xu et al., 2018), and neuroinflammation could be an important modulator of these behavioral alterations. Palmitic acid abolished the migration and phagocytic activity of microglia in response to interferon-γ, thus affecting the protective response of these cells after an inflammatory challenge *in vitro* (Yanguas-Casás et al., 2018). Post-mortem analysis of brain tissue from MDD patients indicated a 6.5% increase in palmitic acid and a 6.2% decrease in oleic acid in the amygdala, as compared to controls (Hamazaki et al., 2012), further suggesting that altered levels of specific fatty acids may be implicated in brain dysfunction.

One potential mechanistic connection between neuroinflammation and mood disorders is the positive effect of pro-inflammatory cytokines on microglial expression of indolamine-2,3-dioxygenase (IDO), the enzyme that converts tryptophan to kynurenine (Wichers and Maes, 2004; Dantzer et al., 2008). Lower availability of tryptophan in the brain due to upregulation of this alternative pathway could slow down its conversion to 5-hydroxytryptophan, the rate-limiting step in serotonin synthesis, carried out by tryptophan hydroxylase. Notably, while far from the only factor involved, serotonin depletion has been shown to induce depressive-like symptoms in animal models and impact mood in humans under certain conditions (Ruhé et al., 2007; O’Connor et al., 2009). Furthermore, increased kynurenine metabolism may result in excessive production of 3-hydroxykynurenine, a generator of reactive oxygen species (ROS), and quinolinic acid, an NMDA receptor agonist, both of which could have their own implications to depression (Müller and Schwarz, 2007).

Another possible mechanism linking neuroinflammation to mood involves precisely the vulnerability of monoaminergic pathways to oxidative stress. Tetrahydrobiopterin (BH_4_) is an essential cofactor, required for certain enzymatic reactions such as those carried out by tryptophan hydroxylase, phenylalanine hydroxylase (which coverts phenylalanine to tyrosine) and tyrosine hydroxylase (which converts tyrosine to L-DOPA, the rate limiting step in dopamine synthesis). BH_4_ may be readily inactivated by ROS, a likely event in strong proinflammatory contexts, thus affecting dopamine and serotonin levels (reviewed by Swardfager et al., 2016). Notably, in addition to the role of serotonin mentioned above, recent reports have shown that dopamine neurotransmission, particularly in the ventral tegmental area-nucleus accumbens circuit, is essential for the expression of depressed phenotypes and social behavior, and thus its depletion could contribute to mood disorders (Tye et al., 2012; Gunaydin et al., 2014; Matthews et al., 2016).

## Polyunsaturated Fatty Acids, Neuroinflammation and Links to Mood Disorders

The nutritional transition observed worldwide in the past few decades has introduced high amounts of SFAs and omega-6 (n-6) PUFAs in the human diet through increased intake of dairy products, vegetable oils and red meat. This change in dietary profile was further accompanied by a reduction in consumption of fruits, vegetables, legumes, grains and fish, important sources of omega-3 (n-3) PUFAs. These changes resulted in an increase in omega-6/omega-3 ratio from about 1:1 to 10:1, reaching up to 20–25:1 or higher, and an alarming omega-3 deficiency in the global population, mainly in Western countries (Simopoulos, 2011).

Omega-3 and omega-6 PUFAs are categorized in these two groups according to the position of the double bond closest to the methyl terminus of the hydrocarbon chain, and, together, comprise the very-long chain family of polyunsaturated fatty acids (VLC-PUFAs). The main VLC-PUFAs in humans are the omega-3 PUFAs, EPA and DHA, and the omega-6 PUFA, ARA, which are components of membrane phospholipids and important signaling molecules (Zárate et al., 2017). In humans, VLC-PUFAs are endogenously synthesized in small amounts from dietary intake of the essential fatty acids, linoleic acid (LA) and alpha-linolenic acid (ALA). These are precursors of ARA, EPA, and DHA synthesis through the action of elongase and desaturase enzymes, which successively elongate and include double bonds into the carbon chain. Thus, adequate balance of these nutrients in the diet is necessary for healthy development, survival and aging (Calder, 2018).

The brain is a lipid-rich organ, and approximately 35% of those lipids are PUFAs (Yehuda et al., 1999). DHA and ARA are major PUFA components in brain cells. They are predominantly found esterified as glycerophospholipids at the plasma membrane (approximately 10,000 nmol per gram of brain tissue) but are also found at much lower amounts in non-esterified form (about 1 nmol per gram of brain tissue). They act as structural components and signaling molecules in neurons, glial cells, and endothelial cells (Bazinet and Layé, 2014). Studies in humans and, mainly, in animal models have revealed that PUFAs enter the brain via lipoproteins or albumin transport in esterified form, as lysophosphatidylcholine, or in non-esterified form, by passive diffusion through a flip-flop mechanism or through protein transporters, such as fatty acid binding proteins (FABPs), fatty acid transport protein (FATP), fatty acid translocases (FAT/CD36) and major facilitator superfamily domain-containing protein 2 (Mfsd2a) (Lauritzen et al., 2001; Umhau et al., 2009; Domenichiello et al., 2014; Nguyen et al., 2014; Chen et al., 2015; Liu et al., 2015; Pan et al., 2015, 2016; Hachem et al., 2016). PUFAs play important roles in brain function, including synaptic plasticity, neurotransmission, metabolism, neurogenesis, neuroinflammation and neuroprotection (Bazinet and Layé, 2014). Not surprisingly, therefore, reduced or unbalanced dietary supply and brain levels of PUFAs (notably, DHA) are associated with brain disorders, including cognitive and mood disorders (see below).

In addition to modulation of serotonin (5-HT1 and 5-HT4), beta-adrenergic and dopamine (D1 and D2) receptor signaling through increased adenylate cyclase and protein kinase A (PKA) activities (Liu et al., 2015), PUFAs play an important role in neuroinflammation, an important etiologic factor of mood disorders (Chang et al., 2015; Yirmiya et al., 2015; Chen et al., 2018). Omega-6 and omega-3 PUFAs have opposite effects on inflammatory modulation. ARA is an important precursor of eicosanoids, bioactive molecules that regulate the inflammatory process in immune cells. In response to inflammatory stimuli, membrane phospholipids are cleaved by phospholipase A2 (PLA-2) and release ARA, a substrate of cyclooxygenase (COX), lipoxygenase (LOX) and cytochrome P450. This stimulates synthesis of prostaglandins (PGs), thromboxanes (TXs), and leukotrienes (LTs), key pro-inflammatory mediators (Innes and Calder, 2018). Post-mortem analysis of brains from patients with bipolar disorders indicated a dysregulation of ARA release and downstream metabolism in frontal cortex (Kim et al., 2009), and mood stabilizers such as lithium, valproate and carbamazepine have been found to modify the ARA cascade in the brain (Kim et al., 2009). These findings suggest that increased levels of ARA from the diet could lead to exacerbation and dysregulation of the inflammatory response in brain cells, thus contributing to mechanisms associated with mood disorders.

*In vitro* studies showed that omega-3 PUFAs modulate microglial functions. For instance, EPA treatment inhibited microglial production of proinflammatory cytokines (IL-1β, IL-6, and TNF-α) (Liuzzi et al., 2007) *in vitro*, and supplementation with omega-3 PUFAs inhibited microglial activation and shifted microglial profile from the so-called classical pro-inflammatory M1 to the neuroprotective M2 phenotype in a model of brain injury in rats (Chen et al., 2018). When incorporated into microglial membranes, DHA, which has been described as a potent immunomodulator in brain cells (Antonietta Ajmone-Cat et al., 2012), blocked the recognition of LPS by cell surface receptors and inhibited nuclear factor kappa B (NF-κB) activation and synthesis of IL-1β and TNF-α (De Smedt-Peyrusse et al., 2008). In addition, DHA prevented LPS-induced neuroinflammation and restored synaptic structure and functions in hippocampal CA1 pyramidal neurons (Chang et al., 2015). In Fat-1 mice, which convert n-6 to n-3 PUFAs in the brain, feeding with a DHA-enriched diet prevented LPS-induced increases in pro-inflammatory cytokines, microglial activation, depressive-like behavior and reduction in BDNF levels (Gu et al., 2018).

Omega-3 PUFAs, mainly EPA, are competitive substrates for enzymes involved in the biosynthesis of inflammatory mediators derived from ARA. Increased PUFA consumption results in a membrane phospholipid composition with increased levels of these fatty acids, and in a reduction of ARA-derived inflammatory mediators (reviewed in Calder, 2015). Moreover, DHA and EPA are precursors of important lipid mediators with anti-inflammatory and pro-resolutive actions, such as resolvins and protectins. Resolvin D1 (RvD1) and resolvin E1 (RvE1), for example, decrease LPS-induced microglial expression of proinflammatory cytokines, namely TNF-α, IL-6, and IL-1β (Rey et al., 2016).

## Fatty Acids, Microbiota Changes, and Mood Disorders

Recently, the gut–microbiota–brain axis has been implicated in neuroinflammation and the development of neuropsychiatric disorders. A comparative analysis between children from a rural African village in Burkina Faso (fed a rural diet) and European children (fed a modern Western diet) indicated significant differences in gut microbiota between the two groups (De Filippo et al., 2010), and suggested an important role of the nutritional transition in altering the human gut microbiome and in the development of inflammatory diseases.

The gut microbiome rapidly responds to dietary composition. Using mouse models, David et al. showed that short-term exposure to diets enriched in animal or plant products changed microbiota composition and microbial gene expression (David et al., 2014). Feeding a HFD caused shifts in the gut bacterial ecosystem in mice (Daniel et al., 2014). More recently, mice fed a HFD for 8 weeks were shown to present a depressive-like phenotype accompanied by a relative reduction in the population of Bacteroidetes and increase in the population of Firmicutes and Cyanobacteria in their caecal microbiome (Hassan et al., 2018). Interestingly, MDD patients showed different abundances of Firmicutes, Actinobacteria and Bacteroidetes when compared to healthy controls. In the same study, transplantation of fecal microbiota from MDD patients into mice resulted in depressive-like behaviors compared with colonization with microbiota derived from healthy control individuals (Zheng et al., 2016). Similarly, transplantation of fecal microbiota from depressed patients to microbiota-depleted rats induced anhedonia and anxiety-like behaviors (Kelly et al., 2016).

The detailed mechanisms underlying how changes in microbiota may lead to mood disorders remain unclear, but neuroinflammation appears as a potential mechanism. Microglia from germ-free mice showed decreased expression of genes associated with inflammation and defense responses, and an immature profile when compared with microglia from control mice (Matcovitch-Natan et al., 2016; Fung et al., 2017). Moreover, microbiota complexity has a central role in microglia function, regulating the neuroinflammatory response in health and disease (Erny et al., 2015).

## Adiponectin

Adiponectin, a hormone released by adipocytes and found abundantly in plasma and at lower concentrations in the CSF (Ebinuma et al., 2007), has been linked to mood disorders, and may connect dietary changes to behavior, particularly with respect to long-term effects. Circulating levels of adiponectin and the response elicited by activation of its receptors, AdipoR1 and AdipoR2 (found in several organs, including the brain), have been shown to be modulated by inflammatory and metabolic conditions, such as obesity and diabetes (Hotta et al., 2000; Yang et al., 2002). Although consistent human data are lacking, adiponectin has anti-depressant (Liu et al., 2012) and anti-inflammatory (Ouchi and Walsh, 2007) properties in mice. It has also been shown to be a candidate mediator of the positive effects of exercise and environmental enrichment on neurogenesis, mood, and cognition (Chabry et al., 2015).

## The Insulin-Dopamine Link

Kleinridders et al. (2015) showed that reduced insulin signaling in the brain, as a result of insulin resistance, led to increased levels of monoamine oxidases and increased dopamine clearance. They further showed that this change in dopamine metabolism led to age-related anxiety and depressive-like behavior in mice, results consistent with the above mentioned increasingly important role of dopamine signaling in mood disorders.

Complementing their previous results (Kleinridders et al., 2015), the same group later used conditional insulin receptor knockout mice to show that insulin signaling in astrocytes has a role in regulating dopaminergic transmission, via the release of the gliotransmitter ATP (Cai et al., 2018). Their results suggest that activation of insulin receptors in astrocytes activates Munc18c to promote ATP exocitosis, which acts on P2X receptors on dopaminergic neurons to modulate dopamine release and normal mood behavior. These results also led to the conclusion that dopamine signaling may be altered and contribute to mood disorders in an insulin resistance scenario (Cai et al., 2018).

Moreover, Fordahl and Jones (2017) demonstrated in mice that prolonged consumption of an HFD impairs insulin signaling in the nucleus accumbens and reduces dopamine reuptake in dopaminergic terminals. Notably, restoring insulin signaling could revert this deficit, suggesting that loss of insulin sensitivity may be the cause of altered dopaminergic in the region (Fordahl and Jones, 2017).

## Perspectives and Concluding Remarks

**FIGURE 1:**
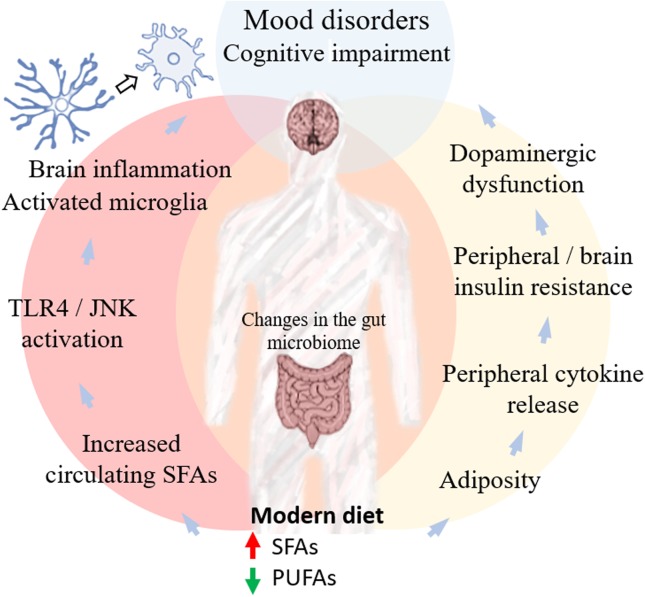
Pathways linking diet and mental health. The nutritional transition observed in modern society, mainly in Western countries, has resulted in increased consumption of SFAs and reduced intake of PUFAs. Excessive energy intake from fat-enriched diets increases fatty acid storage and surpasses the remodeling capacity of adipose tissue, resulting in macrophage recruitment and increasing circulating levels of proinflammatory cytokines. Increased cytokine levels lead to the activation of stress kinases, such as JNK and IKKβ, resulting in increased IRS-1 serine phosphorylation and reduced insulin-stimulated PI3-kinase activity, causing central and peripheral insulin resistance. Brain inflammation and insulin resistance have been implicated in cognitive deficits (reviewed in De Felice et al., 2014; Ferreira et al., 2014). A recent report further suggested that reduced insulin signaling in the brain affects dopamine metabolism and release, contributing to mood disorders (Cai et al., 2018). Whether this dopaminergic dysfunction is also related to cognitive impairment is a possibility yet unexplored. Excessive energy intake from dietary fat further results in dysregulation of free fatty acid (FFA) fluxes, reducing PUFAs and increasing SFAs in the circulation. Although the exact mechanism is still controversial (Lancaster et al., 2018), several reports have shown that SFAs activate TLR4-dependent signaling pathways that increase inflammatory responses in microglia and induce brain inflammation, another potential mechanism involved in the development of both mood disorders and cognitive impairment. At the same time, dietary choices are known to impact the gut microbiota, which may regulate neuroinflammatory responses. Together, these interconnecting mechanisms suggest relevant links between lipid imbalance associated with fat-enriched diets and the onset and progression of neuropsychiatric and cognitive disorders.

Given the high rate of failure of antidepressant therapies, with at least 30% of patients being unresponsive to multiple rounds of pharmacological treatment (Sinyor et al., 2010), and the lack of effective, disease modifying treatments for dementia, the prospect of dietary interventions for mood and cognitive disorders is appealing. Notably, the targets involved in potential dietary approaches to mental health may in fact overlap with targets for pharmacotherapy in current clinical trials, including neuroinflammation (e.g., TLR and cytokine receptors) and brain insulin signaling (Figure 1). To reach this goal, an important step will be to understand and dissect the distinct but interdependent roles of fatty acids as nutrients and signaling molecules in the brain, and their impact on brain function and dysfunction. Finally, since no food is consumed in isolation by humans, this should happen as part of a larger effort to explore the already proposed potential of other nutrients, particularly carbohydrates, as competing players in both inflammation and insulin signaling (DiNicolantonio et al., 2018).

## Author Contributions

All authors discussed and contributed ideas to the manuscript. HM and LS shared most of the writing work. SF advised the process and edited the manuscript.

## Conflict of Interest Statement

The authors declare that the research was conducted in the absence of any commercial or financial relationships that could be construed as a potential conflict of interest.

## References

[B1] Antonietta Ajmone-Cat M. Lavinia Salvatori M. De Simone R. Mancini M. Biagioni S. Bernardo A. (2012). Docosahexaenoic acid modulates inflammatory and antineurogenic functions of activated microglial cells. *J. Neurosci. Res.* 90 575–587. 10.1002/jnr.22783 22057807

[B2] Arcego D. M. Toniazzo A. P. Krolow R. Lampert C. Berlitz C. dos Santos Garcia E. (2018). Impact of high-fat diet and early stress on depressive-like behavior and hippocampal plasticity in adult male rats. *Mol. Neurobiol.* 55 2740–2753. 10.1007/s12035-017-0538-y 28451885

[B3] Arkan M. C. Hevener A. L. Greten F. R. Maeda S. Li Z. W. Long J. M. (2005). IKK-β links inflammation to obesity-induced insulin resistance. *Nat. Med.* 11 191–198. 10.1038/nm1185 15685170

[B4] Barberger-Gateau P. Raffaitin C. Letenneur L. Berr C. Tzourio C. Dartigues J. F. (2007). Dietary patterns and risk of dementia: the Three-City cohort study. *Neurology* 69 1921–1930. 10.1212/01.wnl.0000278116.37320.52 17998483

[B5] Barberger-Gateau P. Samieri C. Feart C. Plourde M. (2011). Dietary omega 3 polyunsaturated fatty acids and Alzheimers disease: interaction with apolipoprotein E genotype. *Curr. Alzheimer Res.* 8 479–491. 10.2174/156720511796391926 21605054 PMC3518784

[B6] Barnard N. D. Bunner A. E. Agarwal U. (2014). Saturated and trans fats and dementia: a systematic review. *Neurobiol. Aging* 35 S65–S73. 10.1016/J.NEUROBIOLAGING.2014.02.030 24916582

[B7] Barnes A. S. (2011). The epidemic of obesity and diabetes: trends and treatments. *Texas Hear. Inst. J.* 38 142–144. PMC3066828 21494521

[B8] Bazinet R. P. Layé S. (2014). Polyunsaturated fatty acids and their metabolites in brain function and disease. *Nat. Rev. Neurosci.* 15 771–785. 10.1038/nrn3820 25387473

[B9] Beattie E. C. Stellwagen D. Morishita W. Bresnahan J. C. Ha B. K. Von Zastrow M. (2002). Control of synaptic strength by glial TNFalpha. *Science* 295 2282–2285. 10.1126/science.1067859 11910117

[B10] Berger M. E. Smesny S. Kim S. W. Davey C. G. Rice S. Sarnyai Z. (2017). Omega-6 to omega-3 polyunsaturated fatty acid ratio and subsequent mood disorders in young people with at-risk mental states: a 7-year longitudinal study. *Transl. Psychiatry* 7:e1220. 10.1038/tp.2017.190 28850110 PMC5611753

[B11] Berthoud H.-R. (2012). The neurobiology of food intake in an obesogenic environment. *Proc. Natl. Acad. Sci. U.S.A.* 71 478–487. 10.1017/S0029665112000602 22800810 PMC3617987

[B12] Bray G. A. Nielsen S. J. Popkin B. M. (2004). Consumption of high-fructose corn syrup in beverages may play a role in the epidemic of obesity. *Am. J. Clin. Nutr.* 79 537–543. 10.1093/ajcn/79.4.537 15051594

[B13] Bray G. A. Popkin B. M. (1998). Dietary fat intake does affect obesity! *Am. J. Clin. Nutr.* 68 1157–1173. 10.1093/ajcn/68.6.1157 9846842

[B14] Bray G. A. Popkin B. M. (2014). Dietary sugar and body weight: have we reached a crisis in the epidemic of obesity and diabetes: health be damned! Pour on the sugar. *Diabetes Care* 37 950–956. 10.2337/dc13-2085 24652725 PMC9514031

[B15] Buettner R. Schölmerich J. Bollheimer L. C. (2007). High-fat diets: modeling the metabolic disorders of human obesity in rodents. *Obesity* 15 798–808. 10.1038/oby.2007.608 17426312

[B16] Cai W. Xue C. Sakaguchi M. Konishi M. Shirazian A. Ferris H. A. (2018). Insulin regulates astrocyte gliotransmission and modulates behavior. *J. Clin. Invest.* 128 2914–2926. 10.1172/JCI99366 29664737 PMC6025980

[B17] Calabrese F. Rossetti A. C. Racagni G. Gass P. Riva M. A. Molteni R. (2014). Brain-derived neurotrophic factor: a bridge between inflammation and neuroplasticity. *Front. Cell. Neurosci.* 8:430. 10.3389/fncel.2014.00430 25565964 PMC4273623

[B18] Calder P. C. (2015). Marine omega-3 fatty acids and inflammatory processes: effects, mechanisms and clinical relevance. *Biochim. Biophys. Acta* 1851 469–484. 10.1016/j.bbalip.2014.08.010 25149823

[B19] Calder P. C. (2018). Very long-chain n-3 fatty acids and human health: fact, fiction and the future. *Proc. Nutr. Soc.* 77 52–72. 10.1017/S0029665117003950 29039280

[B20] Chabry J. Nicolas S. Cazareth J. Murris E. Guyon A. Glaichenhaus N. (2015). Enriched environment decreases microglia and brain macrophages inflammatory phenotypes through adiponectin-dependent mechanisms: relevance to depressive-like behavior. *Brain Behav. Immun.* 50 275–287. 10.1016/j.bbi.2015.07.018 26209808

[B21] Chang P. Khatchadourian A. McKinney R. Maysinger D. (2015). Docosahexaenoic acid (DHA): a modulator of microglia activity and dendritic spine morphology. *J. Neuroinflammation* 12:34. 10.1186/s12974-015-0244-5 25889069 PMC4344754

[B22] Chen C. T. Kitson A. P. Hopperton K. E. Domenichiello A. F. Trépanier M.-O. Lin L. E. (2015). Plasma non-esterified docosahexaenoic acid is the major pool supplying the brain. *Sci. Rep.* 5:15791. 10.1038/srep15791 26511533 PMC4625162

[B23] Chen X. Chen C. Fan S. Wu S. Yang F. Fang Z. (2018). Omega-3 polyunsaturated fatty acid attenuates the inflammatory response by modulating microglia polarization through SIRT1-mediated deacetylation of the HMGB1/NF-κB pathway following experimental traumatic brain injury. *J. Neuroinflammation* 15:116. 10.1186/s12974-018-1151-3 29678169 PMC5909267

[B24] Curb J. D. Marcus E. B. (1991). Body fat and obesity in Japanese Americans. *Am. J. Clin. Nutr.* 53 1552S–1555S. 10.1093/ajcn/53.6.1552S 2031486

[B25] Daniel H. Gholami A. M. Berry D. Desmarchelier C. Hahne H. Loh G. (2014). High-fat diters gut microbiota physiology in mice. *ISME J.* 8 295–308. 10.1038/ismej.2013.155 24030595 PMC3906816

[B26] Dantzer R. O’Connor J. C. Freund G. G. Johnson R. W. Kelley K. W. (2008). From inflammation to sickness and depression: when the immune system subjugates the brain. *Nat. Rev. Neurosci.* 9 46–56. 10.1038/nrn2297 18073775 PMC2919277

[B27] David L. A. Maurice C. F. Carmody R. N. Gootenberg D. B. Button J. E. Wolfe B. E. (2014). Diet rapidly and reproducibly alters the human gut microbiome. *Nature* 505 559–563. 10.1038/nature12820 24336217 PMC3957428

[B28] Davis J. E. Gabler N. K. Walker-Daniels J. Spurlock M. E. (2008). Tlr-4 deficiency selectively protects against obesity induced by diets high in saturated fat. *Obesity* 16 1248–1255. 10.1038/oby.2008.210 18421279

[B29] De Felice F. G. (2013). Alzheimer’s disease and insulin resistance: translating basic science into clinical applications. *J. Clin. Invest.* 123 531–539. 10.1172/JCI64595 23485579 PMC3561831

[B30] De Felice F. G. Ferreira S. T. (2014). Inflammation, defective insulin signaling, and mitochondrial dysfunction as common molecular denominators connecting type 2 diabetes to Alzheimer disease. *Diabetes* 63 2262–2272. 10.2337/db13-1954 24931033

[B31] De Felice F. G. Lourenco M. V. Ferreira S. T. (2014). How does brain insulin resistance develop in Alzheimer’s disease? *Alzheimers Dement.* 10 S26–S32. 10.1016/j.jalz.2013.12.004 24529521

[B32] De Filippo C. Cavalieri D. Paola M. Di Ramazzotti M. Poullet J. B. (2010). Impact of diet in shaping gut microbiota revealed by a comparative study in children from Europe and rural Africa. *Proc. Natl. Acad. Sci. U.S.A.* 107 14691–14696. 10.1073/PNAS.1005963107 20679230 PMC2930426

[B33] De Smedt-Peyrusse V. Sargueil F. Moranis A. Harizi H. Mongrand S. Layé S. (2008). Docosahexaenoic acid prevents lipopolysaccharide-induced cytokine production in microglial cells by inhibiting lipopolysaccharide receptor presentation but not its membrane subdomain localization. *J. Neurochem.* 105 296–307. 10.1111/j.1471-4159.2007.05129.x 18021297

[B34] DiNicolantonio J. J. Mehta V. Onkaramurthy N. O’Keefe J. H. (2018). Fructose-induced inflammation and increased cortisol: a new mechanism for how sugar induces visceral adiposity. *Prog. Cardiovasc. Dis.* 61 3–9. 10.1016/j.pcad.2017.12.001 29225114

[B35] Domenichiello A. F. Chen C. T. Trepanier M.-O. Stavro P. M. Bazinet R. P. (2014). Whole body synthesis rates of DHA from α-linolenic acid are greater than brain DHA accretion and uptake rates in adult rats. *J. Lipid Res.* 55 62–74. 10.1194/jlr.M042275 24212299 PMC3927474

[B36] Dresner A. Laurent D. Marcucci M. Griffin M. E. Dufour S. Cline G. W. (1999). Effects of free fatty acids on glucose transport and IRS-1–associated phosphatidylinositol 3-kinase activity. *J. Clin. Invest.* 103 253–259. 10.1172/JCI5001 9916137 PMC407880

[B37] Dutheil S. Ota K. T. Wohleb E. S. Rasmussen K. Duman R. S. (2016). High-fat diet induced anxiety and anhedonia: impact on brain homeostasis and inflammation. *Neuropsychopharmacology* 41 1874–1887. 10.1038/npp.2015.357 26658303 PMC4869056

[B38] Ebinuma H. Miida T. Yamauchi T. Hada Y. Hara K. Kubota N. (2007). Improved ELISA for selective measurement of adiponectin multimers and identification of adiponectin in human cerebrospinal fluid. *Clin. Chem.* 53 1541–1544. 10.1373/clinchem.2007.085654 17599956

[B39] Eder K. Baffy N. Falus A. Fulop A. K. (2009). The major inflammatory mediator interleukin-6 and obesity. *Inflamm. Res.* 58 727–736. 10.1007/s00011-009-0060-4 19543691

[B40] Erny D. Hrabě de Angelis A. L. Jaitin D. Wieghofer P. Staszewski O. David E. (2015). Host microbiota constantly control maturation and function of microglia in the CNS. *Nat. Neurosci.* 18 965–977. 10.1038/nn.4030 26030851 PMC5528863

[B41] Erridge C. Samani N. J. (2009). Saturated fatty acids do not directly stimulate toll-like receptor signaling. *Arterioscler. Thromb. Vasc. Biol.* 29 1944–1949. 10.1161/ATVBAHA.109.194050 19661481

[B42] Ferreira S. T. Clarke J. R. Bomfim T. R. De Felice F. G. (2014). Inflammation, defective insulin signaling, and neuronal dysfunction in Alzheimer’s disease. *Alzheimers Dement.* 10 S76–S83. 10.1016/j.jalz.2013.12.010 24529528

[B43] Fessler M. B. Rudel L. L. Brown J. M. (2009). Toll-like receptor signaling links dietary fatty acids to the metabolic syndrome. *Curr. Opin. Lipidol.* 20 379–385. 10.1097/MOL.0b013e32832fa5c4 19625959 PMC3099529

[B44] Fordahl S. C. Jones S. R. (2017). High-fat-diet-induced deficits in dopamine terminal function are reversed by restoring insulin signaling. *ACS Chem. Neurosci.* 8 290–299. 10.1021/acschemneuro.6b00308 27966885 PMC5789793

[B45] Fung T. C. Olson C. A. Hsiao E. Y. (2017). Interactions between the microbiota, immune and nervous systems in health and disease. *Nat. Neurosci.* 20 145–155. 10.1038/nn.4476 28092661 PMC6960010

[B46] Goodyear L. J. Giorgino F. Sherman L. A. Carey J. Smith R. J. Dohm G. L. (1995). Insulin receptor phosphorylation, insulin receptor substrate-1 phosphorylation, and phosphatidylinositol 3-kinase activity are decreased in intact skeletal muscle strips from obese subjects. *J. Clin. Invest.* 95 2195–2204. 10.1172/JCI117909 7537758 PMC295829

[B47] Greenberg A. S. Obin M. S. (2006). Obesity and the role of adipose tissue in inflammation and metabolism. *Am. J. Clin. Nutr.* 83 461S–465S. 10.1093/ajcn/83.2.461S 16470013

[B48] Gu M. Li Y. Tang H. Zhang C. Li W. Zhang Y. (2018). Endogenous omega (N)-3 fatty acids in fat-1 mice attenuated depression-like behavior, imbalance between microglial M1 and M2 phenotypes, and dysfunction of neurotrophins induced by lipopolysaccharide administration. *Nutrients* 10:E1351. 10.3390/nu10101351 30248907 PMC6213921

[B49] Gunaydin L. A. Grosenick L. Finkelstein J. C. Kauvar I. V. Fenno L. E. Adhikari A. (2014). Natural neural projection dynamics underlying social behavior. *Cell* 157 1535–1551. 10.1016/j.cell.2014.05.017 24949967 PMC4123133

[B50] Hachem M. Géloën A. Van A. L. Foumaux B. Fenart L. Gosselet F. (2016). Efficient docosahexaenoic acid uptake by the brain from a structured phospholipid. *Mol. Neurobiol.* 53 3205–3215. 10.1007/s12035-015-9228-9 26041661

[B51] Hagnell O. (1989). Repeated incidence and prevalence studies of mental disorders in a total population followed during 25 years The Lundby Study, Sweden. *Acta Psychiatr. Scand.* 79 61–77. 10.1111/j.1600-0447.1989.tb05216.x 2801180

[B52] Hamazaki K. Hamazaki T. Inadera H. (2012). Fatty acid composition in the postmortem amygdala of patients with schizophrenia, bipolar disorder, and major depressive disorder. *J. Psychiatr. Res.* 46 1024–1028. 10.1016/J.JPSYCHIRES.2012.04.012 22572570

[B53] Hariri N. Thibault L. (2010). High-fat diet-induced obesity in animal models. *Nutr. Res. Rev.* 23 270–299. 10.1017/S0954422410000168 20977819

[B54] Hassan A. M. Mancano G. Kashofer K. Fröhlich E. E. Matak A. Mayerhofer R. (2018). High-fat diet induces depression-like behaviour in mice associated with changes in microbiome, neuropeptide Y, and brain metabolome. *Nutr. Neurosci.* 10.1080/1028415X.2018.1465713 [Epub ahead of print]. 29697017

[B55] Heyward F. D. Walton R. G. Carle M. S. Coleman M. A. Garvey W. T. Sweatt J. D. (2012). Adult mice maintained on a high-fat diet exhibit object location memory deficits and reduced hippocampal SIRT1 gene expression. *Neurobiol. Learn. Mem.* 98 25–32. 10.1016/J.NLM.2012.04.005 22542746 PMC3389577

[B56] Hidaka B. H. (2012). Depression as a disease of modernity: explanations for increasing prevalence. *J. Affect. Disord.* 140 205–214. 10.1016/j.jad.2011.12.036 22244375 PMC3330161

[B57] Hill J. O. Peters J. C. (1998). Environmental contributions to the obesity epidemic. *Science* 280 1371–1374. 10.1126/SCIENCE.280.5368.1371 9603719

[B58] Hirosumi J. Tuncman G. Chang L. Görgün C. Z. Uysal K. T. Maeda K. (2002). A central role for JNK in obesity and insulin resistance. *Nature* 420 333–336. 10.1038/nature01137 12447443

[B59] Holt R. I. G. de Groot M. Golden S. H. (2014). Diabetes and depression. *Curr. Diab. Rep.* 14:491. 10.1007/s11892-014-0491-3 24743941 PMC4476048

[B60] Horton T. J. Drougas H. Brachey A. Reed G. W. Peters J. C. Hill J. O. (1995). Fat and carbohydrate overfeeding in humans: different effects on energy storage. *Am. J. Clin. Nutr.* 62 19–29. 10.1093/ajcn/62.1.19 7598063

[B61] Hotamisligil G. S. Shargill N. S. Spiegelman B. M. (1993). Adipose expression of tumor necrosis factor-alpha: direct role in obesity-linked insulin resistance. *Science* 259 87–91. 7678183 10.1126/science.7678183

[B62] Hotta K. Funahashi T. Arita Y. Takahashi M. Matsuda M. Okamoto Y. (2000). Plasma concentrations of a novel, adipose-specific protein, adiponectin, in type 2 diabetic patients. *Arterioscler. Thromb. Vasc. Biol.* 20 1595–1599. 10845877 10.1161/01.atv.20.6.1595

[B63] Hu S. Wang L. Yang D. Li L. Togo J. Wu Y. (2018). Dietary fat, but not protein or carbohydrate, regulates energy intake and causes adiposity in mice. *Cell Metab.* 28 415–431.e4. 10.1016/j.cmet.2018.06.010 30017356

[B64] Huang S. Rutkowsky J. M. Snodgrass R. G. Ono-Moore K. D. Schneider D. A. Newman J. W. (2012). Saturated fatty acids activate TLR-mediated proinflammatory signaling pathways. *J. Lipid Res.* 53 2002–2013. 10.1194/jlr.D029546 22766885 PMC3413240

[B65] Innes J. K. Calder P. C. (2018). Omega-6 fatty acids and inflammation. *Prostaglandins Leukot. Essent. Fatty Acids* 132 41–48. 10.1016/J.PLEFA.2018.03.004 29610056

[B66] Jacka F. N. (2017). Nutritional psychiatry: where to next? *EBioMedicine* 17 24–29. 10.1016/j.ebiom.2017.02.020 28242200 PMC5360575

[B67] Jacka F. N. Pasco J. A. Mykletun A. Williams L. J. Hodge A. M. O’Reilly S. L. (2010). Association of western and traditional diets with depression and anxiety in women. *Am. J. Psychiatry* 167 305–311. 10.1176/appi.ajp.2009.09060881 20048020

[B68] Jeon B. T. Jeong E. A. Shin H. J. Lee Y. Lee D. H. Kim H. J. (2012). Resveratrol attenuates obesity-associated peripheral and central inflammation and improves memory deficit in mice fed a high-fat diet. *Diabetes* 61 1444–1454. 10.2337/db11-1498 22362175 PMC3357272

[B69] Kahn R. Sievenpiper J. L. (2014). Dietary sugar and body weight: have we reached a crisis in the epidemic of obesity and diabetes? We have, but the pox on sugar is overwrought and overworked. *Diabetes Care* 37 957–962. 10.2337/dc13-2506 24652726

[B70] Kalmijn S. Launer L. J. Ott A. Witteman J. C. M. Hofman A. Breteler M. M. B. (1997). Dietary fat intake and the risk of incident dementia in the Rotterdam study. *Ann. Neurol.* 42 776–782. 10.1002/ana.410420514 9392577

[B71] Kelly J. R. Borre Y. O’ Brien C. Patterson E. El Aidy S. Deane J. (2016). Transferring the blues: depression-associated gut microbiota induces neurobehavioural changes in the rat. *J. Psychiatr. Res.* 82 109–118. 10.1016/j.jpsychires.2016.07.019 27491067

[B72] Khan T. A. Sievenpiper J. L. (2016). Controversies about sugars: results from systematic reviews and meta-analyses on obesity, cardiometabolic disease and diabetes. *Eur. J. Nutr.* 55 25–43. 10.1007/s00394-016-1345-3 27900447 PMC5174149

[B73] Kim H.-W. Rapoport S. I. Rao J. S. (2009). Altered arachidonic acid cascade enzymes in postmortem brain from bipolar disorder patients. *Mol. Psychiatry* 16 419–428. 10.1038/mp.2009.137 20038946 PMC3190400

[B74] Kleinert M. Clemmensen C. Hofmann S. M. Moore M. C. Renner S. Woods S. C. (2018). Animal models of obesity and diabetes mellitus. *Nat. Rev. Endocrinol.* 14 140–162. 10.1038/nrendo.2017.161 29348476

[B75] Kleinridders A. Cai W. Cappellucci L. Ghazarian A. Collins W. R. Vienberg S. G. (2015). Insulin resistance in brain alters dopamine turnover and causes behavioral disorders. *Proc. Natl. Acad. Sci. U.S.A.* 112 3463–3468. 10.1073/pnas.1500877112 25733901 PMC4371978

[B76] Kopelman P. G. (2000). Obesity as a medical problem. *Nature* 404 635–643. 10.1038/35007508 10766250

[B77] Kreutzberg G. W. (1996). Microglia: a sensor for pathological events in the CNS. *Trends Neurosci.* 19 312–318. 10.1016/0166-2236(96)10049-7 8843599

[B78] Krishnan V. Nestler E. J. (2011). Animal models of depression: molecular perspectives. *Curr. Top. Behav. Neurosci.* 7 121–147. 10.1007/7854_2010_108 21225412 PMC3270071

[B79] Lai J. S. Hiles S. Bisquera A. Hure A. J. McEvoy M. Attia J. (2014). A systematic review and meta-analysis of dietary patterns and depression in community-dwelling adults. *Am. J. Clin. Nutr.* 99 181–197. 10.3945/ajcn.113.069880 24196402

[B80] Laitinen M. H. Ngandu T. Rovio S. Helkala E.-L. Uusitalo U. Viitanen M. (2006). Fat intake at midlife and risk of dementia and Alzheimer’s disease: a population-based study. *Dement. Geriatr. Cogn. Disord.* 22 99–107. 10.1159/000093478 16710090

[B81] Lancaster G. I. Langley K. G. Berglund N. A. Kammoun H. L. Reibe S. Estevez E. (2018). Evidence that TLR4 is not a receptor for saturated fatty acids but mediates lipid-induced inflammation by reprogramming macrophage metabolism. *Cell Metab.* 27 1096–1110.e5. 10.1016/j.cmet.2018.03.014 29681442

[B82] Lauritzen L. Hansen H. Jørgensen M. Michaelsen K. (2001). The essentiality of long chain n-3 fatty acids in relation to development and function of the brain and retina. *Prog. Lipid Res.* 40 1–94. 10.1016/S0163-7827(00)00017-5 11137568

[B83] Lawton C. L. Delargy H. J. Brockman J. Smith F. C. Blundell J. E. (2000). The degree of saturation of fatty acids influences post-ingestive satiety. *Br. J. Nutr.* 83 473–482. 10953671

[B84] Lê K.-A. Ith M. Kreis R. Faeh D. Bortolotti M. Tran C. (2009). Fructose overconsumption causes dyslipidemia and ectopic lipid deposition in healthy subjects with and without a family history of type 2 diabetes. *Am. J. Clin. Nutr.* 89 1760–1765. 10.3945/ajcn.2008.27336 19403641

[B85] Lee J. Y. Sohn K. H. Rhee S. H. Hwang D. (2001). Saturated fatty acids, but not unsaturated fatty acids, induce the expression of cyclooxygenase-2 mediated through toll-like receptor 4. *J. Biol. Chem.* 276 16683–16689. 10.1074/jbc.M011695200 11278967

[B86] Lee Y. S. Li P. Huh J. Y. Hwang I. J. Lu M. Kim J. I. (2011). Inflammation is necessary for long-term but not short-term high-fat diet-induced insulin resistance. *Diabetes* 60 2474–2483. 10.2337/db11-0194 21911747 PMC3178297

[B87] Lin P.-Y. Huang S.-Y. Su K.-P. (2010). A meta-analytic review of polyunsaturated fatty acid compositions in patients with depression. *Biol. Psychiatry* 68 140–147. 10.1016/j.biopsych.2010.03.018 20452573

[B88] Liu J. Guo M. Zhang D. Cheng S.-Y. Liu M. Ding J. (2012). Adiponectin is critical in determining susceptibility to depressive behaviors and has antidepressant-like activity. *Proc. Natl. Acad. Sci. U.S.A.* 109 12248–12253. 10.1073/pnas.1202835109 22778410 PMC3409774

[B89] Liu J. J. Green P. John Mann J. Rapoport S. I. Sublette M. E. (2015). Pathways of polyunsaturated fatty acid utilization: implications for brain function in neuropsychiatric health and disease. *Brain Res.* 1597 220–246. 10.1016/j.brainres.2014.11.059 25498862 PMC4339314

[B90] Liuzzi G. M. Latronico T. Rossano R. Viggiani S. Fasano A. Riccio P. (2007). Inhibitory effect of polyunsaturated fatty acids on MMP-9 release from microglial cells–implications for complementary multiple sclerosis treatment. *Neurochem. Res.* 32 2184–2193. 10.1007/s11064-007-9415-9 17624613

[B91] Logan A. C. Jacka F. N. (2014). Nutritional psychiatry research: an emerging discipline and its intersection with global urbanization, environmental challenges and the evolutionary mismatch. *J. Physiol. Anthropol.* 33:22. 10.1186/1880-6805-33-22 25060574 PMC4131231

[B92] Lu Y.-C. Yeh W.-C. Ohashi P. S. (2008). LPS/TLR4 signal transduction pathway. *Cytokine* 42 145–151. 10.1016/J.CYTO.2008.01.006 18304834

[B93] Luchsinger J. A. (2012). Type 2 diabetes and cognitive impairment: linking mechanisms. *J. Alzheimers Dis.* 30 S185–S198. 10.3233/JAD-2012-111433 22433668 PMC3372666

[B94] Ludwig D. S. Peterson K. E. Gortmaker S. L. (2001). Relation between consumption of sugar-sweetened drinks and childhood obesity: a prospective, observational analysis. *Lancet* 357 505–508. 10.1016/S0140-6736(00)04041-1 11229668

[B95] Ludwig D. S. Willett W. C. Volek J. S. Neuhouser M. L. (2018). Dietary fat: from foe to friend? *Science* 770 764–770. 10.1126/science.aau2096 30442800

[B96] Lumeng C. N. Bodzin J. L. Saltiel A. R. (2007). Obesity induces a phenotypic switch in adipose tissue macrophage polarization. *J. Clin. Invest.* 117 175–184. 10.1172/JCI29881 17200717 PMC1716210

[B97] Malik V. S. Popkin B. M. Bray G. A. Després J.-P. Willett W. C. Hu F. B. (2010). Sugar-sweetened beverages and risk of metabolic syndrome and type 2 diabetes: a meta-analysis. *Diabetes Care* 33 2477–2483. 10.2337/dc10-1079 20693348 PMC2963518

[B98] Malik V. S. Schulze M. B. Hu F. B. (2006). Intake of sugar-sweetened beverages and weight gain: a systematic review. *Am. J. Clin. Nutr.* 84 274–288. 10.1093/ajcn/84.2.274 16895873 PMC3210834

[B99] Matcovitch-Natan O. Winter D. R. Giladi A. Vargas Aguilar S. Spinrad A. Sarrazin S. (2016). Microglia development follows a stepwise program to regulate brain homeostasis. *Science* 353:aad8670. 10.1126/science.aad8670 27338705

[B100] Matthews G. A. Nieh E. H. Vander Weele C. M. Halbert S. A. Pradhan R. V. Yosafat A. S. (2016). Dorsal raphe dopamine neurons represent the experience of social isolation. *Cell* 164 617–631. 10.1016/j.cell.2015.12.040 26871628 PMC4752823

[B101] McNamara R. K. Hahn C.-G. Jandacek R. Rider T. Tso P. Stanford K. E. (2007). Selective deficits in the omega-3 fatty acid docosahexaenoic acid in the postmortem orbitofrontal cortex of patients with major depressive disorder. *Biol. Psychiatry* 62 17–24. 10.1016/j.biopsych.2006.08.026 17188654

[B102] McNamara R. K. Jandacek R. Tso P. Dwivedi Y. Ren X. Pandey G. N. (2013). Lower docosahexaenoic acid concentrations in the postmortem prefrontal cortex of adult depressed suicide victims compared with controls without cardiovascular disease. *J. Psychiatr. Res.* 47 1187–1191. 10.1016/j.jpsychires.2013.05.007 23759469 PMC3710518

[B103] Mechawar N. Savitz J. (2016). Neuropathology of mood disorders: do we see the stigmata of inflammation? *Transl. Psychiatry* 6:e946. 10.1038/tp.2016.212 27824355 PMC5314124

[B104] Mock K. Lateef S. Benedito V. A. Tou J. C. (2017). High-fructose corn syrup-55 consumption alters hepatic lipid metabolism and promotes triglyceride accumulation. *J. Nutr. Biochem.* 39 32–39. 10.1016/J.JNUTBIO.2016.09.010 27768909

[B105] Mocking R. J. T. Harmsen I. Assies J. Koeter M. W. J. Ruhé H. G. Schene A. H. (2016). Meta-analysis and meta-regression of omega-3 polyunsaturated fatty acid supplementation for major depressive disorder. *Transl. Psychiatry* 6:e756. 10.1038/tp.2016.29 26978738 PMC4872453

[B106] Montonen J. Järvinen R. Knekt P. Heliövaara M. Reunanen A. (2007). Consumption of sweetened beverages and intakes of fructose and glucose predict type 2 diabetes occurrence. *J. Nutr.* 137 1447–1454. 10.1093/jn/137.6.1447 17513405

[B107] Morris M. C. Evans D. A. Bienias J. L. Tangney C. C. Bennett D. A. Aggarwal N. (2003a). Dietary fats and the risk of incident Alzheimer disease. *Arch. Neurol.* 60 194–200. 12580703 10.1001/archneur.60.2.194

[B108] Morris M. C. Evans D. A. Bienias J. L. Tangney C. C. Bennett D. A. Wilson R. S. (2003b). Consumption of fish and n-3 fatty acids and risk of incident Alzheimer disease. *Arch. Neurol.* 60 940–946. 10.1001/archneur.60.7.940 12873849

[B109] Morris M. C. Evans D. A. Bienias J. L. Tangney C. C. Wilson R. S. (2004). Dietary fat intake and 6-year cognitive change in an older biracial community population. *Neurology* 62 1573–1579. 10.1212/01.WNL.0000123250.82849.B6 15136684

[B110] Mosca A. Nobili V. De Vito R. Crudele A. Scorletti E. Villani A. (2017). Serum uric acid concentrations and fructose consumption are independently associated with NASH in children and adolescents. *J. Hepatol.* 66 1031–1036. 10.1016/j.jhep.2016.12.025 28214020

[B111] Moussavi N. Gavino V. Receveur O. (2008). Could the quality of dietary fat, and not just its quantity, be related to risk of obesity? *Obesity* 16 7–15. 10.1038/oby.2007.14 18223605

[B112] Müller N. Schwarz M. J. (2007). The immune-mediated alteration of serotonin and glutamate: towards an integrated view of depression. *Mol. Psychiatry* 12 988–1000. 10.1038/sj.mp.4002006 17457312

[B113] Nguyen L. N. Ma D. Shui G. Wong P. Cazenave-Gassiot A. Zhang X. (2014). MFSD2A is a transporter for the essential omega-3 fatty acid docosahexaenoic acid. *Nature* 509 503–506. 10.1038/nature13241 24828044

[B114] O’Connor J. C. André C. Wang Y. Lawson M. A. Szegedi S. S. Lestage J. (2009). Interferon-gamma and tumor necrosis factor-alpha mediate the upregulation of indoleamine 2,3-dioxygenase and the induction of depressive-like behavior in mice in response to bacillus Calmette-Guerin. *J. Neurosci.* 29 4200–4209. 10.1523/JNEUROSCI.5032-08.2009 19339614 PMC2835569

[B115] O’Neil A. Quirk S. E. Housden S. Brennan S. L. Williams L. J. Pasco J. A. (2014). Relationship between diet and mental health in children and adolescents: a systematic review. *Am. J. Public Health* 104 e31–e42. 10.2105/AJPH.2014.302110 25208008 PMC4167107

[B116] Ortega R. M. Requejo A. M. Andrés P. López-Sobaler A. M. Quintas M. E. Redondo M. R. (1997). Dietary intake and cognitive function in a group of elderly people. *Am. J. Clin. Nutr.* 66 803–809. 10.1093/ajcn/66.4.803 9322553

[B117] Ott A. Stolk R. P. Hofman A. Van Harskamp F. Grobbee D. E. Breteler M. M. B. (1996). Association of diabetes mellitus and dementia: the Rotterdam study. *Diabetologia* 39 1392–1397. 10.1007/s001250050588 8933010

[B118] Ouchi N. Walsh K. (2007). Adiponectin as an anti-inflammatory factor. *Clin. Chim. Acta* 380 24–30. 10.1016/j.cca.2007.01.026 17343838 PMC2755046

[B119] Ouyang X. Cirillo P. Sautin Y. McCall S. Bruchette J. L. Diehl A. M. (2008). Fructose consumption as a risk factor for non-alcoholic fatty liver disease. *J. Hepatol.* 48 993–999. 10.1016/j.jhep.2008.02.011 18395287 PMC2423467

[B120] Pal D. Dasgupta S. Kundu R. Maitra S. Das G. Mukhopadhyay S. (2012). Fetuin-A acts as an endogenous ligand of TLR4 to promote lipid-induced insulin resistance. *Nat. Med.* 18 1279–1285. 10.1038/nm.2851 22842477

[B121] Pan Y. Scanlon M. J. Owada Y. Yamamoto Y. Porter C. J. H. Nicolazzo J. A. (2015). Fatty acid-binding protein 5 facilitates the blood–brain barrier transport of docosahexaenoic acid. *Mol. Pharm.* 12 4375–4385. 10.1021/acs.molpharmaceut.5b00580 26455443

[B122] Pan Y. Short J. L. Choy K. H. C. Zeng A. X. Marriott P. J. Owada Y. (2016). Fatty acid-binding protein 5 at the blood-brain barrier regulates endogenous brain docosahexaenoic acid levels and cognitive function. *J. Neurosci.* 36 11755–11767. 10.1523/JNEUROSCI.1583-16.2016 27852782 PMC6705637

[B123] Paolicelli R. C. Bolasco G. Pagani F. Maggi L. Scianni M. Panzanelli P. (2011). Synaptic pruning by microglia is necessary for normal brain development. *Science* 333 1456–1458. 10.1126/science.1202529 21778362

[B124] Park B. S. Song D. H. Kim H. M. Choi B.-S. Lee H. Lee J.-O. (2009). The structural basis of lipopolysaccharide recognition by the TLR4–MD-2 complex. *Nature* 458 1191–1195. 10.1038/nature07830 19252480

[B125] Phillips C. M. Kesse-Guyot E. McManus R. Hercberg S. Lairon D. Planells R. (2012). High dietary saturated fat intake accentuates obesity risk associated with the fat mass and obesity–associated gene in adults. *J. Nutr.* 142 824–831. 10.3945/jn.111.153460 22457394

[B126] Piers L. S. Walker K. Z. Stoney R. M. Soares M. J. O’Dea K. (2003). Substitution of saturated with monounsaturated fat in a 4-week diet affects body weight and composition of overweight and obese men. *Br. J. Nutr.* 90 717–727. 10.1079/BJN2003948 13129479

[B127] Poggi M. Bastelica D. Gual P. Iglesias M. A. Gremeaux T. Knauf C. (2007). C3H/HeJ mice carrying a toll-like receptor 4 mutation are protected against the development of insulin resistance in white adipose tissue in response to a high-fat diet. *Diabetologia* 50 1267–1276. 10.1007/s00125-007-0654-8 17426960

[B128] Popkin B. M. Adair L. S. Ng S. W. (2012). Global nutrition transition and the pandemic of obesity in developing countries. *Nutr. Rev.* 70 3–21. 10.1111/j.1753-4887.2011.00456.x 22221213 PMC3257829

[B129] Popkin B. M. Gordon-Larsen P. (2004). The nutrition transition: worldwide obesity dynamics and their determinants. *Int. J. Obes.* 28 2–9. 10.1038/sj.ijo.0802804 15543214

[B130] Psaltopoulou T. Sergentanis T. N. Panagiotakos D. B. Sergentanis I. N. Kosti R. Scarmeas N. (2013). Mediterranean diet, stroke, cognitive impairment, and depression: a meta-analysis. *Ann. Neurol.* 74 580–591. 10.1002/ana.23944 23720230

[B131] Rebolledo-Solleiro D. Roldán-Roldán G. Díaz D. Velasco M. Larqué C. Rico-Rosillo G. (2017). Increased anxiety-like behavior is associated with the metabolic syndrome in non-stressed rats. *PLoS One* 12:e0176554. 10.1371/journal.pone.0176554 28463967 PMC5413000

[B132] Reichelt A. C. Westbrook R. F. Morris M. J. (2017). Editorial: impact of diet on learning, memory and cognition. *Front. Behav. Neurosci.* 11:96. 10.3389/fnbeh.2017.00096 28579950 PMC5437154

[B133] Rey C. Nadjar A. Buaud B. Vaysse C. Aubert A. Pallet V. (2016). Resolvin D1 and E1 promote resolution of inflammation in microglial cells in vitro. *Brain Behav. Immun.* 55 249–259. 10.1016/J.BBI.2015.12.013 26718448

[B134] Roden M. Price T. B. Perseghin G. Petersen K. F. Rothman D. L. Cline G. W. (1996). Mechanism of free fatty acid-induced insulin resistance in humans. *J. Clin. Invest.* 97 2859–2865. 10.1172/JCI118742 8675698 PMC507380

[B135] Rogero M. M. Calder P. C. (2018). Obesity, inflammation, toll-like receptor 4 and fatty acids. *Nutrients* 10:E432. 10.3390/nu10040432 29601492 PMC5946217

[B136] Rosqvist F. Iggman D. Kullberg J. Cedernaes J. Johansson H. E. Larsson A. (2014). Overfeeding polyunsaturated and saturated fat causes distinct effects on liver and visceral fat accumulation in humans. *Diabetes* 63 2356–2368. 10.2337/db13-1622 24550191

[B137] Ruhé H. G. Mason N. S. Schene A. H. (2007). Mood is indirectly related to serotonin, norepinephrine and dopamine levels in humans: a meta-analysis of monoamine depletion studies. *Mol. Psychiatry* 12 331–359. 10.1038/sj.mp.4001949 17389902

[B138] Santos L. E. Beckman D. Ferreira S. T. (2016). Microglial dysfunction connects depression and Alzheimer’s disease. *Brain Behav. Immun.* 55 151–165. 10.1016/j.bbi.2015.11.011 26612494

[B139] Scarmeas N. Stern Y. Tang M.-X. Mayeux R. Luchsinger J. A. (2006). Mediterranean diet and risk for Alzheimer’s disease. *Ann. Neurol.* 59 912–921. 10.1002/ana.20854 16622828 PMC3024594

[B140] Sevilla-González M. del R. Quintana-Mendoza B. M. Aguilar-Salinas C. A. (2017). Interaction between depression, obesity, and type 2 diabetes: a complex picture. *Arch. Med. Res.* 48 582–591. 10.1016/j.arcmed.2018.02.004 29478673

[B141] Sheline Y. I. (2011). Depression and the hippocampus: cause or effect? *Biol. Psychiatry* 70 308–309. 10.1016/j.biopsych.2011.06.006 21791257 PMC3733566

[B142] Shi H. Kokoeva M. V. Inouye K. Tzameli I. Yin H. Flier J. S. (2006). TLR4 links innate immunity and fatty acid-induced insulin resistance. *J. Clin. Invest.* 116 3015–3025. 10.1172/JCI28898 17053832 PMC1616196

[B143] Simopoulos A. P. (2011). Evolutionary aspects of diet: the omega-6/omega-3 ratio and the brain. *Mol. Neurobiol.* 44 203–215. 10.1007/s12035-010-8162-0 21279554

[B144] Singh A. Abraham W. C. (2017). Astrocytes and synaptic plasticity in health and disease. *Exp. Brain Res.* 235 1645–1655. 10.1007/s00221-017-4928-1 28299411

[B145] Sinyor M. Schaffer A. Levitt A. (2010). The sequenced treatment alternatives to relieve depression (STAR^∗^D) trial: a review. *Can. J. Psychiatry* 55 126–135. 10.1177/070674371005500303 20370962

[B146] Spiegelman B. M. Flier J. S. (2001). Obesity and the regulation review of energy balance. *Cell* 104 531–543. 11239410 10.1016/s0092-8674(01)00240-9

[B147] Stanhope K. L. (2016). Sugar consumption, metabolic disease and obesity: the state of the controversy. *Crit. Rev. Clin. Lab. Sci.* 53 52–67. 10.3109/10408363.2015.1084990 26376619 PMC4822166

[B148] Stanhope K. L. Schwarz J. M. Keim N. L. Griffen S. C. Bremer A. A. Graham J. L. (2009). Consuming fructose-sweetened, not glucose-sweetened, beverages increases visceral adiposity and lipids and decreases insulin sensitivity in overweight/obese humans. *J. Clin. Invest.* 119 1322–1334. 10.1172/JCI37385 19381015 PMC2673878

[B149] Sun K. Kusminski C. M. Scherer P. E. (2011). Adipose tissue remodeling and obesity. *J. Clin. Invest.* 121 2094–2101. 10.1172/JCI45887 21633177 PMC3104761

[B150] Swardfager W. Rosenblat J. D. Benlamri M. McIntyre R. S. (2016). Mapping inflammation onto mood: inflammatory mediators of anhedonia. *Neurosci. Biobehav. Rev.* 64 148–166. 10.1016/j.neubiorev.2016.02.017 26915929

[B151] Tye K. M. Mirzabekov J. J. Warden M. R. Ferenczi E. A. Tsai H.-C. Finkelstein J. (2012). Dopamine neurons modulate neural encoding and expression of depression-related behaviour. *Nature* 493 537–541. 10.1038/nature11740 23235822 PMC4160519

[B152] Umhau J. C. Zhou W. Carson R. E. Rapoport S. I. Polozova A. Demar J. (2009). Imaging incorporation of circulating docosahexaenoic acid into the human brain using positron emission tomography. *J. Lipid Res.* 50 1259–1268. 10.1194/jlr.M800530-JLR200 19112173 PMC2694326

[B153] Vagena E. Ryu J. K. Baeza-Raja B. Walsh N. M. Syme C. Day J. P. (2018). A high-fat diet promotes depression-like behavior in mice by suppressing hypothalamic PKA signaling. *SSRN Electron. J.* 10.2139/ssrn.3188483 PMC6510753 31076569

[B154] Valdearcos M. Robblee M. M. Benjamin D. I. Nomura D. K. Xu A. W. Koliwad S. K. (2014). Microglia dictate the impact of saturated fat consumption on hypothalamic inflammation and neuronal function. *Cell Rep.* 9 2124–2138. 10.1016/j.celrep.2014.11.018 25497089 PMC4617309

[B155] van Dijk S. J. Feskens E. J. M. Bos M. B. Hoelen D. W. M. Heijligenberg R. Bromhaar M. G. (2009). A saturated fatty acid-rich diet induces an obesity-linked proinflammatory gene expression profile in adipose tissue of subjects at risk of metabolic syndrome. *Am. J. Clin. Nutr.* 90 1656–1664. 10.3945/ajcn.2009.27792.INTRODUCTION 19828712

[B156] Vergnaud A.-C. Norat T. Mouw T. Romaguera D. May A. M. Bueno-de-Mesquita H. B. (2013). Macronutrient composition of the diet and prospective weight change in participants of the EPIC-PANACEA study. *PLoS One* 8:e57300. 10.1371/journal.pone.0057300 23472080 PMC3589445

[B157] Vessby B. Uusitupa M. Hermansen K. Riccardi G. Rivellese A. A. Tapsell L. C. (2001). Substituting dietary saturated for monounsaturated fat impairs insulin sensitivity in healthy men and women: the KANWU study. *Diabetologia* 44 312–319. 10.1007/s001250051620 11317662

[B158] Wang H. Zhou J. Liu Q. Z. Wang L. L. Shang J. (2017). Simvastatin and bezafibrate ameliorate emotional disorder induced by high fat diet in C57BL/6 mice. *Sci. Rep.* 7:2335. 10.1038/s41598-017-02576-5 28539670 PMC5443827

[B159] Wang Z. Liu D. Wang F. Liu S. Zhao S. Ling E. A. (2012). Saturated fatty acids activate microglia via Toll-like receptor 4/NF-κB signalling. *Br. J. Nutr.* 107 229–241. 10.1017/S0007114511002868 21733316

[B160] Wellen K. E. Hotamisligil G. S. (2003). Obesity-induced inflammatory changes in adipose tissue. *J. Clin. Invest.* 112 1785–1788. 10.1172/JCI20514 14679172 PMC297006

[B161] Wichers M. C. Maes M. (2004). The role of indoleamine 2,3-dioxygenase (IDO) in the pathophysiology of interferon-alpha-induced depression. *J. Psychiatry Neurosci.* 29 11–17. 14719046 PMC305266

[B162] Willett W. C. (1998). Is dietary fat a major determinant of body fat? *Am. J. Clin. Nutr.* 67 556S–562S. 10.1093/ajcn/67.3.556S 9497170

[B163] Willett W. C. (2002). Dietary fat plays a major role in obesity: no. *Obes. Rev.* 3 59–68. 10.1046/j.1467-789X.2002.00060.x 12120421

[B164] Xu H. Barnes G. T. Yang Q. Tan G. Yang D. Chou C. J. (2003). Chronic inflammation in fat plays a crucial role in the development of obesity-related insulin resistance. *J. Clin. Invest.* 112 1821–1830. 10.1172/JCI19451 14679177 PMC296998

[B165] Xu L. Xu S. Lin L. Gu X. Fu C. Fang Y. (2018). High-fat diet mediates anxiolytic-like behaviors in a time-dependent manner through the regulation of SIRT1 in the brain. *Neuroscience* 372 237–245. 10.1016/j.neuroscience.2018.01.001 29331532

[B166] Yang J. L. Liu D. X. Jiang H. Pan F. Ho C. S. Ho R. C. M. (2016). The effects of high-fat-diet combined with chronic unpredictable mild stress on depression-like behavior and leptin/LepRb in male rats. *Sci. Rep.* 6:35239. 10.1038/srep35239 27739518 PMC5064321

[B167] Yang W.-S. Lee W.-J. Funahashi T. Tanaka S. Matsuzawa Y. Chao C.-L. (2002). Plasma adiponectin levels in overweight and obese Asians. *Obes. Res.* 10 1104–1110. 10.1038/oby.2002.150 12429873

[B168] Yanguas-Casás N. Crespo-Castrillo A. de Ceballos M. L. Chowen J. A. Azcoitia I. Arevalo M. A. (2018). Sex differences in the phagocytic and migratory activity of microglia and their impairment by palmitic acid. *Glia* 66 522–537. 10.1002/glia.23263 29139169

[B169] Yehuda S. Rabinovitz S. Mostofsky D. I. (1999). Essential fatty acids are mediators of brain biochemistry and cognitive functions. *J. Neurosci. Res.* 56 565–570. 10.1002/(SICI)1097-4547(19990615)56:6<565::AID-JNR2>3.0.CO;2-H 10374811

[B170] Yirmiya R. Rimmerman N. Reshef R. (2015). Depression as a microglial disease. *Trends Neurosci.* 38 637–658. 10.1016/j.tins.2015.08.001 26442697

[B171] Yuan M. Konstantopoulos N. Lee J. Hansen L. Li Z. W. Karin M. (2001). Reversal of obesity- and diet-induced insulin resistance with salicylates or targeted disruption of Ikkbeta. *Science* 293 1673–1677. 10.1126/science.1061620 11533494

[B172] Zárate R. El Jaber-Vazdekis N. Tejera N. Pérez J. A. Rodríguez C. (2017). Significance of long chain polyunsaturated fatty acids in human health. *Clin. Transl. Med.* 6:25. 10.1186/s40169-017-0153-6 28752333 PMC5532176

[B173] Zheng P. Zeng B. Zhou C. Liu M. Fang Z. Xu X. (2016). Gut microbiome remodeling induces depressive-like behaviors through a pathway mediated by the host’s metabolism. *Mol. Psychiatry* 21 786–796. 10.1038/mp.2016.44 27067014

